# Current Challenges in the Management of Sepsis and Septic Shock: Personalized, Physiology-Guided Treatment

**DOI:** 10.3390/jcm11154565

**Published:** 2022-08-04

**Authors:** Athanasios Chalkias

**Affiliations:** 1 Department of Anesthesiology, Faculty of Medicine, University of Thessaly, 41500 Larisa, Greece; thanoschalkias@yahoo.gr; 2 Outcomes Research Consortium, Cleveland, OH 44195, USA

Sepsis is life-threatening organ dysfunction caused by a dysregulated host response to infection affecting millions of people each year. Sepsis is present in more than 50% of adult hospitalizations in the United States and is an immediate cause of death in approximately 17% of these cases [1]. The consequences of the sepsis syndrome are so severe that the World Health Assembly and the WHO made sepsis a global health priority in 2017. Septic patients have a higher risk for secondary organ injury, especially those with critical illnesses, and therefore, early identification and appropriate management are crucial for improving outcomes.

During recent decades, a substantial amount of research has improved the clinical management of septic patients. Anesthesiologists and intensivists had central roles in this evolution and continue to become involved in the identification and management of patients with sepsis and septic shock. Despite progress in anesthesia and intensive care, and mounting evidence, these clinical entities are associated with substantial short- and long-term morbidity and high mortality [1,2].

Several scientific organizations have developed and updated global guidelines for the management of sepsis and septic shock, trying to address the challenges of treating patients with advanced disease. Nevertheless, many recommendations remain controversial. Additionally, large randomized controlled trials may contribute to strengthening the evidence but do not always help us improve patient management.

The septic syndrome begins with an infection that initiates a host inflammatory response. This is a straightforward and easily understood first step. A highly complex series of events that may involve all organs and every system within the body then quickly unfolds. Targeted interventions are more successful before patients reach this critical point, after which the underlying pathophysiology is more severe and medical treatment may not be effective. Standard therapeutic efforts attempt to support organ perfusion and function, to control the source of infection as soon as possible, and to modulate the host response.

Current sepsis-care bundles may be considered practical or achievable [2], but one size does not fit all patients, as their heterogeneity and clinical phenotypes greatly reduce the effectiveness of evidence-based interventions. This is further complicated by the inter-patient variability in response to treatment, which has increased efforts towards to the understanding of the genetic basis of sepsis [3,4]. Pharmacogenomics is another rapidly evolving field, aiming at identifying which septic patients are likely to benefit from or to be harmed by drug treatments. However, in the era of genetic revolution and discoveries in molecular mechanisms of sepsis, the systems’ physiology is often forgotten and sometimes poorly understood [5,6].

Indeed, the management of patients is challenging due to diminished physiological reserves and comorbidities, systemic response to surgery or other invasive procedures, the administration of anesthetics and mechanical ventilation, and/or the availability of resources. Therefore, a question arises: what is the best approach to resuscitation of a patient with sepsis and septic shock? There remains no clear answer due to the lack of solid evidence. However, multimodal perfusion monitoring and an integrated evaluation of macro- and microcirculatory hemodynamic parameters seem to be necessary additions to the other aspects of care [7,8,9]. Furthermore, criteria to assist in the decision of when to stop aggressive treatment must be urgently established, so as to decrease the risk of over-resuscitation. Perhaps now more than ever, it is imperative to understand the full nature of the response to sepsis; to improve diagnostics; and to advance therapies within a personalized, physiology-guided treatment strategy.

Today, the body of evidence continues to grow due to extensive research and thousands of publications each year. Nevertheless, the nature of sepsis and the significant costs required to treat these patients continue to challenge both health care providers and health systems. We are extremely aware that every minute counts when limiting organ dysfunction. If anything, these challenges certainly motivate us to give our best in improving the management of septic patients throughout the continuum of severity, ranging from sepsis to septic shock.
